# Whole Transcriptome Analyses Reveal Differential mRNA and microRNA Expression Profiles in Primary Human Dermal Fibroblasts Infected with Clinical or Vaccine Strains of Varicella Zoster Virus

**DOI:** 10.3390/pathogens8040183

**Published:** 2019-10-10

**Authors:** Soo-Jin Oh, Sooyeon Lim, Moon Jung Song, Jin Hyun Ahn, Chan Hee Lee, Ok Sarah Shin

**Affiliations:** 1 Bk21 PLUS Program, Department of Biomedical Sciences, College of Medicine, Korea University Guro Hospital, Seoul 08308, Korea; sjooooh@gmail.com (S.-J.O.); limsooy@korea.ac.kr (S.L.); 2 Department of Biosystems and Biotechnology, Division of Biotechnology, College of Life Sciences and Biotechnology, Korea University, Seoul 02841, Korea; moonsong@korea.ac.kr; 3 Department of Molecular Cell Biology, Sungkyunkwan University School of Medicine, Suwon 16419, Korea; jahn@skku.edu; 4 Department of Microbiology, Chungbuk National University, Cheongju 28644, Korea; chlee@chungbuk.ac.kr

**Keywords:** Varicella zoster virus, SuduVax, RNA-seq, miRNA

## Abstract

Licensed live attenuated vaccines have been developed to prevent varicella zoster virus (VZV) infection, which causes chickenpox and shingles. The genomic sequences of both clinical- and vaccine-derived VZV strains have been analyzed previously. To further characterize the molecular signatures and complexity of wildtype (clinical) versus attenuated (vaccine-derived) VZV-mediated host cellular responses, we performed high-throughput next generation sequencing to quantify and compare the expression patterns of mRNAs and microRNAs (miRNAs) in primary human dermal fibroblasts (HDFs) infected with wildtype (YC01 low passage) and attenuated (YC01 high passage, SuduVax, and VarilRix) VZV strains. 3D-multidimensional scaling of the differentially expressed genes demonstrated the distinct grouping of wildtype and attenuated strains. In particular, we observed that HDFs infected with attenuated strains had more differentially expressed genes (DEGs) involved in the retinoic-acid inducible gene–I-like receptor and interferon-mediated signaling pathways compared with wildtype strains. Additionally, miRNA expression patterns were profiled following the infection of HDFs with VZV. Small RNA sequencing identified that several miRNAs were upregulated, including miR-146a-5p, which has been associated with other herpesvirus infections, whereas let-7a-3p was downregulated in both wildtype and attenuated VZV-infected cells. This study identified genes and miRNAs that may be essential in VZV pathogenesis.

## 1. Introduction

Varicella zoster virus (VZV), a member of the *Alphaherpesvirinae* family, is the primary causative agent of chickenpox (varicella) [1]. The reactivation of VZV from a latent neuronal state results in shingles (herpes zoster). While varicella presents as a generalized rash with symptoms such as fever and malaise, shingles usually occurs in the elderly and manifests as a painful skin rash [2]. Despite our limited understanding of VZV pathogenesis and immunity, several vaccines have been developed against both varicella and zoster.

The currently available VZV vaccines originate from either the Oka or MAV/06 strains. VarilRix (GSK), VariVax (Merck), and OkaVax (Biken) are derived from Oka strains, whereas the high-dose preparation (19,000 PFU vs. 1350 PFU) ZostaVax (Merck) is routinely used to prevent herpes zoster [3,4]. These vaccines were attenuated from a wildtype virus (pOka) isolated from a small child with primary varicella [5]. Several studies have characterized the single nucleotide polymorphisms of vaccines strains like VarilRix, VariVax, and ZostaVax, and compared them with several clinical strains [6,7,8,9].

The second varicella vaccine strain, MAV/06, was prepared by extensive cellular passages in MRC-5 cells. SuduVax, manufactured by GC Pharma, is an MAV/06-derived vaccine commonly used in Korea. We have previously reported that SuduVax is genetically similar to other Oka-derived vaccines [10,11]. Furthermore, we conducted a comparative genomic analysis of VZV single nucleotide polymorphisms (SNPs) and identified 24 vaccine-specific sites that were commonly found in all vaccine strains, including SuduVax, VariVax, and VarilRix [12]. These nonsynonymous mutations were found in *ORF*s *0*, *6*, *31*, *39*, *55*, *62*, and *64* in all three vaccine strains. Over 40% (9 of 24) of the vaccine-type mutations were found in *ORF62*, which encodes the immediate early protein IE62, a known transactivator of VZV gene expression. Despite the indisputable medical importance, the immunological differences between clinical wildtype VZV strains and attenuated vaccine strains have not yet been fully investigated.

Like other herpes viruses, virulent VZV is known to evade the immune system via several mechanisms, such as impairment of interferon (IFN) expression as well as inhibiting antiviral signaling induced by IFN, downregulation of major histocompatibility complex, and blocking NF-κB signaling [13,14]. Therefore, it is essential to compare the effects of wildtype and attenuated VZV infection to characterize virulence-related immune evasion mechanisms. High-throughput RNA sequencing (RNA-seq), which is an effective method for profiling the transcriptome with high efficiency and accuracy, has recently been used to study the pathogenesis of VZV infection [15,16,17,18,19].

In this study, we used RNA-seq and small RNA-seq to comprehensively analyze whole transcriptomes following wildtype (YC01 low passage) and attenuated (YC01 high passage, SuduVax cell-associated (CA) or cell-free (CF) preparation, and VarilRix) VZV infection. The YC01 strain was isolated from a zoster patient in Korea, whereas vaccine-type mutations were found in highly passaged YC01 strains [20]. Our work provides a global overview of virus type-specific mRNA and miRNA profiles, and gives an insight into the effect of vaccine-type VZV mutations on host immune signaling and the microRNA network.

## 2. Materials and Methods 

### 2.1. Cells and Viruses

Normal human dermal fibroblasts (HDFs) were purchased from Lonza (Basel, Switzerland) and were grown in fibroblast basal medium supplemented with fibroblast growth medium SingleQuots (Lonza). HEK293T and MRC-5 cells (human lung embryonic fibroblasts) were obtained from the ATCC (Manassas, MA, USA) and were grown in DMEM with 10% fetal bovine serum and antibiotics.

The VZV strain YC01 (GenBank Accession No. KJ808816) has been described previously [21] and was propagated in MRC-5 cells. YC01 low passage (YC01-low) and high passage (YC01-high) virus preparation was described in our previous study [20]. SuduVax was sequenced and characterized as described previously [10]. All VZV strains used in this paper were analyzed and genetically characterized by Jeon et al., as reported previously [12]. HDFs were infected with VZV strains at a multiplicity of infection (MOI) of 0.01 in all experiments. Plaque assays were performed to determine the titer of these strains, as described previously [15,19].

### 2.2. RNA-Sequencing

The total RNA of each sample was isolated using TRIzol reagent (Invitrogen, Carlsbad, CA, USA) for RNA sequencing, according to the manufacturer’s instructions. The quality of RNA was checked using a nanodrop and an Agilent 2100 Bioanalyzer (Agilent). All total RNA samples had an RNA Integrity Number (RIN) greater than 9.0, indicating excellent RNA quality. Prior to cDNA library construction, poly (A) mRNA was enriched using 2 µg of total RNA and magnetic beads with an oligo (dT) primer. The purified mRNAs were then disrupted into short fragments, and double-stranded cDNAs were immediately synthesized. Library preparation was carried out as described previously [22]. The cDNAs were subjected to end-repair, poly (A) addition, and connection with sequencing adapters using a TruSeq RNA Sample Prep Kit (Illumina, San Diego, CA, USA). Suitable fragments were automatically purified using a BluePippin 2% agarose gel cassette (Sage Science, Beverly, MA) and were selected as templates for amplification using polymerase chain reaction (PCR). The final library size and quality was evaluated electrophoretically using an Agilent High Sensitivity DNA Kit (Agilent Technologies, Santa Clara, CA), and the fragments were found to be between 350 and 450 bp in size. The library was sequenced using an Illumina HiSeq 2500 sequencer (Illumina, San Diego, CA, USA). Sequencing reads were aligned to the human genome (GenBank accession no. hg19) at all possible positions using Bowtie2 with perfect matches (0 mismatches). The raw RNA-Seq data files were deposited in NCBI’s Gene Expression Omnibus (GEO) and are accessible via the GEO Series accession number GSE121385.

### 2.3. Differentially Expressed Genes (DEGs) and Gene Ontology Analysis 

Gene expression level was measured based on the fragments per kilobase of exon per million mapped reads (FPKM) using Cufflinks v2.1.13 from Ensembl release 72. We generated gene-level count data using HTSeq-count v0.5.4p35, calculated normalization factors using iterative DEGES/edgeR, and filtered DEGs based on a *p*-value < 0.05 and a log_2_ fold change > 2. To characterize the molecular functions of DEGs, we analyzed the gene ontology (GO) (www.geneontology.org). A *p*-value < 0.001 was considered statistically significant. The GO database classifies genes according to three categories (biological process, cellular component, and molecular function) and predicts the function of the selected genes.

### 2.4. Three Dimensional (3D)-Multidimensional Scaling (MDS) 

3D-MDS visualizes proximity data in a way that preserves between-sample distances and is a technique (similar to PCA) commonly used to transform higher-dimension dissimilarity data into a three-dimensional plot. A 3D-MDS representation of samples based on their DEG profiles was used to identify broad, library-wise trends, and was performed using the Bioconductor package edgeR.

### 2.5. Small RNA-seq Library Construction and Sequencing

Small RNA sequencing libraries were constructed using the NEXTflex Small RNA sample preparation protocol with an initial input of 70 ng of total RNA. Adapters were directly and specifically ligated to the microRNA molecules. Firstly, the NEXTflex 3′ 4N Adenylated adapter (5′ rApp /NNNNTGGAATTCTCGGGTGCCAAGG/3ddC/) and the NEXTflex 5′ 4N adapter (5′ GUUCAGAGUUCUACAGUCCGACGAUCNNNN) were ligated to each end of the total RNA samples. The 5′ and 3′ NEXTflex adapter ligated products were then reverse transcribed by M-MuLV reverse transcriptase in the presence of an RNA RT primer (5′ GCCTTGGCACCCGAGAATTCCA) to create single stranded cDNA. The cDNA was then amplified using PCR with a universal primer (5′ AATGATACGGCGACCACCGAGATCTACACGTTCAGAGTTCTACAGTCCGA) and a primer containing barcode sequences for 18 cycles of 20 s at 95 °C, 30 s at 60 °C, and 15 s at 72 °C. The amplified cDNA constructs were separated on a 6% TBE gel (Invitrogen, Carlsbad, CA, USA) and 140–160 bp bands were excised. After gel purification, the cDNA was eluted and concentrated by ethanol precipitation. The quality and size distribution of the adapter-ligated RNAs and amplified libraries were confirmed by electrophoresis on Agilent Bioanalyzer High Sensitivity DNA microfluidic chips (Agilent, Santa Clara, CA, USA). Libraries were quantified using the KAPA Library Quantification Kit (KK4824, Kapa Biosystems, Wilmington, MA, USA) and sequenced using an Illumina HiSeq2500 on rapid run mode. Cluster generation, followed by 2 × 100 cycle sequencing reads separated by paired-end turnaround, was performed using the Illumina HiSeq2500. Images were analyzed using HiSeq Control Software, version 1.8.4.

The overall read qualities, such as “base sequence quality,” “quality score,” “GC content,” “N content,” and “duplication levels” were evaluated using Fastqc [23]. Low-quality bases below Q20 were trimmed from the 3′ ends of each read, and reads shorter than 17 bases were discarded using cutadapt [24]. The high-quality reads were then mapped onto a human reference genome (a version of Ensembl 72 release for humans [25]) using bowtie [26]. The miRNA reads were enumerated using HTSeq [27] with the “intersection-nonempty” mode based on the miRBase 20 release [28]. EdgeR [29] was used to analyze the differential expression between each region for the read counts of each miRNA. Differentially expressed miRNAs were classified using a significance of log_2_ fold change > ±0.3 and a *p*-value < 0.05.

### 2.6. Bioinformatic Prediction of miRNA Targets and Gene Ontology 

The interactive visual analysis tool, miRTarVis+, was used on miRNA–mRNA expression profile data [30]. miRTarVis+ uses prediction algorithms based on both sequence and expression profile data, and supports two most cited sequence-based miRNA target prediction algorithms, TargetScan and microRNA.org.

A list of common gene targets from at least five prediction databases (Tarbase, TargetScan, miRanda, microRNA.org, and microcosm) was obtained for each differentially expressed miRNA, and miRTarVis+ was used to predict the target genes. The GO package in R (http://www.r-project.org/), canonical Kyoto Encyclopedia of Genes and Genomes (KEGG) pathway maps, and Panther protein classification tools were used to annotate miRNA target functions to predict gene ontology (GO) and the biological pathways associated with miRNA targets.

### 2.7. Real-time - Quantitative Polymerase Chain Reaction (RT-qPCR)

RNA-seq results were validated by RT-qPCR on specific target genes. First-strand cDNA was synthesized from 0.5 μg of total RNA using the ImProm-II Reverse Transcription System (Promega, Madison, WI, USA) according to the manufacturer’s instructions with previously described primer sequences [21,26]. The QuantStudio 6 Flex Real-time PCR system (Thermo Fisher Scientific, Waltham, MA, USA) was utilized for cDNA amplification with Power SYBR® Green Master Mix (Invitrogen, Carlsbad, CA, USA) under the following conditions: 95 °C for 10 min, followed by 40 cycles of 95 °C for 30 s and 60 °C for 1 min. Relative mRNA levels were determined using the comparative Ct method, and were normalized against β-actin or Glyceraldehyde 3-phosphate dehydrogenase (GAPDH) level.

RT-qPCR was used to validate miRNA expression changes. miRNAs were isolated from HDFs using a miRNAeasy mini kit (Qiagen, Hilden, Germany), and cDNA were synthesized using a miScript II RT kit (Qiagen, Hilden, Germany) according to the manufacturer’s protocol. miRNAs were detected using specific primers (Qiagen, Hilden, Germany). *SNORD68*, small nuclear RNA, was used as an internal control for normalization. RT-qPCR was performed to detect the levels of specific miRNAs using a miScript SYBR Green PCR kit (Qiagen, Hilden, Germany). Quantification was carried out using a QuantStudio 6 Flex Real-Time PCR System.

### 2.8. Transfection of miRNA Inhibitors

Cells were seeded in six-well plates and incubated for 24 h to attain 80% confluency. Cells were transiently transfected with lipofectamine 2000 and miRNA inhibitors at a final concentration of 10 mM (Bioneer, Daejeon, Korea). Anti-miR miRNA control inhibitor was also used as a negative control. After 24 h, transfection efficiency was measured using RT-qPCR.

### 2.9. Statistical Analysis 

Quantitative data were expressed as the mean ± standard error of the mean (SEM). Statistical analysis was carried out using Graphpad Prism (Graphpad Software, La Jolla, CA, USA), with Student’s *t*-tests performed to compare the controls and the treated groups.

## 3. Results

### 3.1. Analysis of High-Throughput RNA-seq Transcriptome

**Figure 1:**
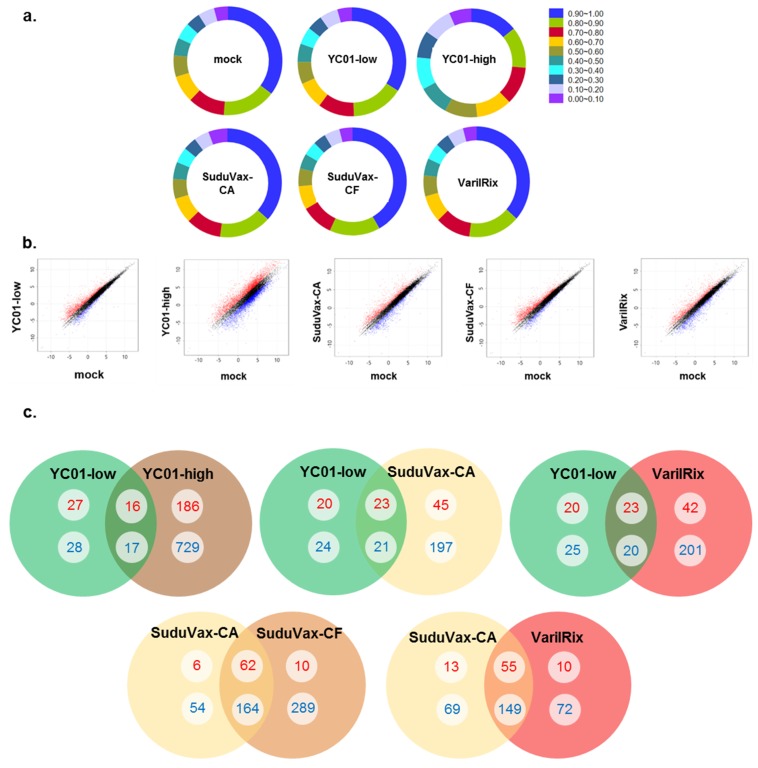
Global overview of the RNA-seq data of wildtype and attenuated strains of varicella zoster virus (VZV). Human dermal fibroblasts (HDFs) were infected with either mock, clinical (YC01 low passage (YC01-low)), vaccine-like (YC01 high passage (YC01-high)), or vaccine (SuduVax cell-associated (CA), SuduVax cell-free (CF), and VarilRix) strains. RNA was harvested 48 h post-infection (hpi). (**a**) The pie chart represents high sequencing coverage in each sample. (**b**) Scatter plot. The x-axis and y-axis indicate the expression levels of genes from mock-infected groups and virus-infected groups, respectively. Red and blue dots represent relatively highly expressed genes in the virus-infected groups and mock-infected groups, respectively. Black dots represent genes that were not classified as differentially expressed. The differentially expressed gene (DEG) count was identified by comparing the mock- and virus-infected groups. (**c**) Venn diagrams showing the overlapping DEG profiles of VZV-infected cells. DEGs have a change of more than 2-fold and a *p*-value of ≤ 0.05. The number of DEGs in the wildtype and attenuated VZV-infected HDFs is given in circles for 2-fold upregulation (red color) and downregulation (blue color).

To achieve global and dynamic host gene expression profiles following VZV infection, we infected human dermal fibroblasts (HDFs) with clinical wildtype (YC01-low) or attenuated (YC01-high, SuduVax cell-associated (CA), SuduVax cell-free (CF), and VarilRix) vaccine strains. RNA-seq was performed to explore the transcriptomes of these infected cells at day 2 post-infection (dpi). Each sample had relatively high sequencing coverage, as presented in the pie charts (Figure 1a). The read depth was distributed relatively evenly along the entire genome, suggesting no introduction of obvious bias during randomly primed reverse transcription and subsequent RNA sequencing.

Differentially expressed genes (DEGs) with a *p*-value < 0.05 and a ±2-fold change in VZV-infected HDFs were identified. A strong correlation was found between the fold change differences and the *p*-values (i.e., genes with a large fold change difference had a low *p*-value in the group-wise comparison). Interestingly, YC01-high VZV-infected cells had the highest number of upregulated and downregulated DEGs compared with the other groups of virus-infected cells (Figure 1b). Venn diagrams were generated to examine the overlap between the mRNA profiles of wildtype and attenuated VZV-infected HDFs (Figure 1c) using the mRNAs that were upregulated or downregulated by at least 2-fold at each time point. The numbers represent the mRNA counts in the indicated area. The upregulated DEG counts are indicated in red, whereas the downregulated DEGs are indicated in blue. There were 16 commonly upregulated DEGs and 17 commonly downregulated DEGs observed in YC01-low and YC01-high VZV-infected HDFs. Interestingly, a higher number of commonly upregulated or downregulated DEGs was observed between attenuated VZV groups (55 commonly upregulated and 149 commonly downregulated DEGs between SuduVax CA and VarilRix). These data indicate a good sample cluster based on gene expression pattern similarity, whilst a clear difference was observed between the wildtype and attenuated VZV-infected HDFs. Moreover, the gene expression profiles were able to discriminate between the two main groups of virus-infected HDFs.

**Figure 2:**
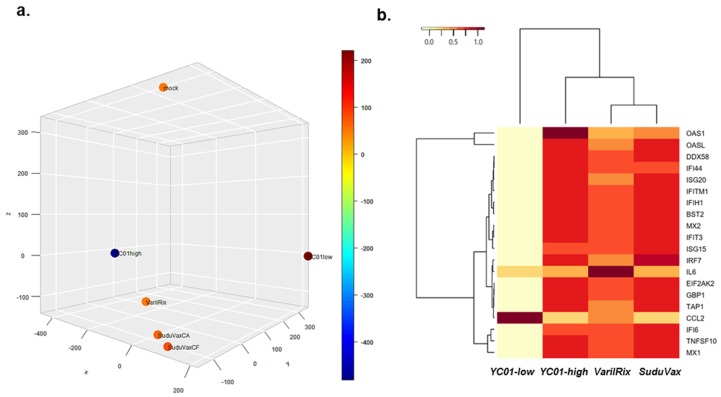
The expression levels of interferon-stimulated genes are distinctly altered by wildtype and attenuated VZV infection. (**a**) 3D-multidimensional scaling (3D-MDS) plot was generated based on all pairwise distances between the global transcriptome-wide RNA-seq profiles of each sample. Wildtype and attenuated VZV-infected samples had a wider distribution than the mock control. (**b**) Heatmaps show the statistical over-representation of the top 20 upregulated DEGs based on the lists of differentially expressed transcripts (compared to the mock control).

To evaluate the degree of proximity between the samples considering biological variations, we compared the wildtype and attenuated VZV-infected human transcriptomes obtained using RNA-seq by performing 3D-MDS (Figure 2a). In general, the wildtype and attenuated VZV-infected samples were distinctly distributed in the 3D-MDS plot and attenuated strains (YC01-high, SuduVax, and VarilRix) were most closely related, suggesting that the samples clustered best according to their respective virus types.

### 3.2. Distinct Changes in Host DEGs in Wildtype and Attenuated VZV-Infected HDFs

To analyze the characteristics of the DEGs, we focused on the 20 DEGs with the most marked upregulation or downregulation in wildtype and attenuated VZV-infected HDFs (Supplementary Table S1). *2*′-*5*′-*Oligoadenylate Synthetase 1(OAS1)* gene expression was upregulated 130-fold in YC01-low-infected cells and more than 508-fold in SuduVax CA-infected cells. A similar pattern was observed for other antiviral genes such as *Mx1*, *Mx2*, *ISG15*, *IFIT1*, *IFIT2*, *IFIT3*, *DDX58*, and *IFIH1*, whose transcript levels were highly elevated. Furthermore, expression levels of *DDX58* and *IFIH1* were highly upregulated in attenuated VZV-infected cells compared with wildtype VZV-infected cells. We also discovered DEGs that were upregulated in virus-infected groups but not in mock-infected groups (Table 1). The genes that were only upregulated in the attenuated VZV-infected groups included *DDX58*, *IFI44*, *IFIH1*, *IFITM1*, and *IL8*.

Figure 2b shows a heatmap of the transcriptomic expression values for the 20 most upregulated DEGs, which were found to be unique to each viral strain. For each specific set of transcripts, hierarchical clustering was performed and is shown as dendrograms. Across all datasets, a heatmap identified the antiviral pathway as a key pathway involving the majority of 20 most upregulated DEGs listed in Supplementary Table S1 (*Mx1*, *Mx2*, *ISG15*, *IFIT1*, *IFIT2*, *IFIT3*, *DDX58*, and *IFIH1*). In addition to virus-sensing pathways, important components of innate immunity and inflammation, such as *IL-6*, were also found to be a part of the key pathways involving the majority of DEGs in all wildtype and attenuated VZV-infected cells, although *IL-6* upregulation was more pronounced in attenuated VZV-infected cells. Presumably, it is possible that as attenuated strains may have lost the functions of virulence factors such as *ORF62*, the virus will no longer interfere with the induction of interferon response.

**Figure 3:**
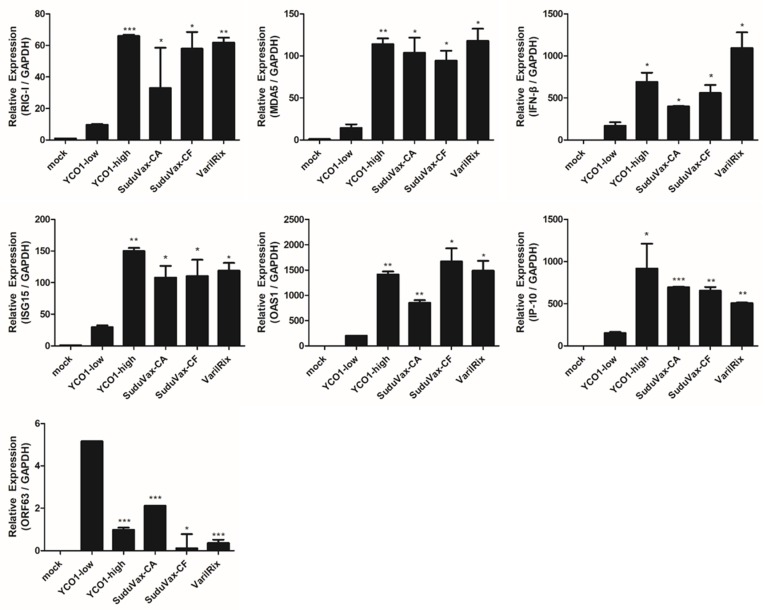
Validation of RNA-seq analysis revealed differential gene expression in VZV-infected cells. HDFs were infected with VZV at a multiplicity of infection (MOI) of 0.01 for 48 h. RT-qPCR was performed to measure *RIG-I*, *MDA5*, *IFN-β*, *ISG-15*, *OAS1*, *IP-10*, and VZV *ORF63* transcript levels. Host and viral gene expression level was calculated relative to the expression level of GAPDH mRNA and is shown as a fold-change relative to the expression levels in mock-infected cells. * *p* < 0.05; ** *p* < 0.01; *** *p* < 0.001, compared with YC01-low-infected cells.

To validate the RNA-sequence analysis, we performed RT-qPCR. Of the mRNAs with distinct expression patterns in wildtype and attenuated VZV strains, we focused on six genes (*DDX58*, *IFIH1*, *IFN-β*, *ISG-15*, *OAS1*, and *IP-10*) for further validation analysis due to their distinct expression in each sample. Higher transcript levels of *DDX58*, *IFIH1*, *IFN-β*, *ISG-15*, *OAS1*, and *IP-10* expression were detected in attenuated VZV-infected cells compared with wildtype VZV-infected cells (Figure 3), suggesting that elevated innate immune activation was induced by attenuated VZV infection, compared with wildtype VZV infection. On the contrary, it was interesting to note that *ORF63* expression was the opposite in that it was higher in wildtype VZV compared with attenuated VZV-infected cells. 

Gene set enrichment analysis using KEGG identified the functional groups significantly enriched by VZV infection. The top 10 upregulated pathways activated in VZV-infected HDFs included the RIG-I-like receptor signaling pathway and the cytosolic DNA-sensing pathway (Supplementary Table S2). Additionally, to examine the biological roles of the DEGs, a gene ontology (GO) enrichment analysis was performed on the upregulated genes. In the biological process GO category, we focused primarily on immune system processes because several genes related to antiviral signaling are included in this category (Supplementary Figure S1). Interestingly, more genes were included in the immune system processes GO category for the attenuated VZV-infected cells than for the wildtype VZV-infected cells. Additionally, we also analyzed viral *ORF* expression patterns, and a heatmap shows distinct changes in VZV *ORF* expression in each viral group (Supplementary Figure S2).

### 3.3. Distinct microRNA Expression Patterns in Response to Wildtype and Attenuated VZV Infection

**Figure 4:**
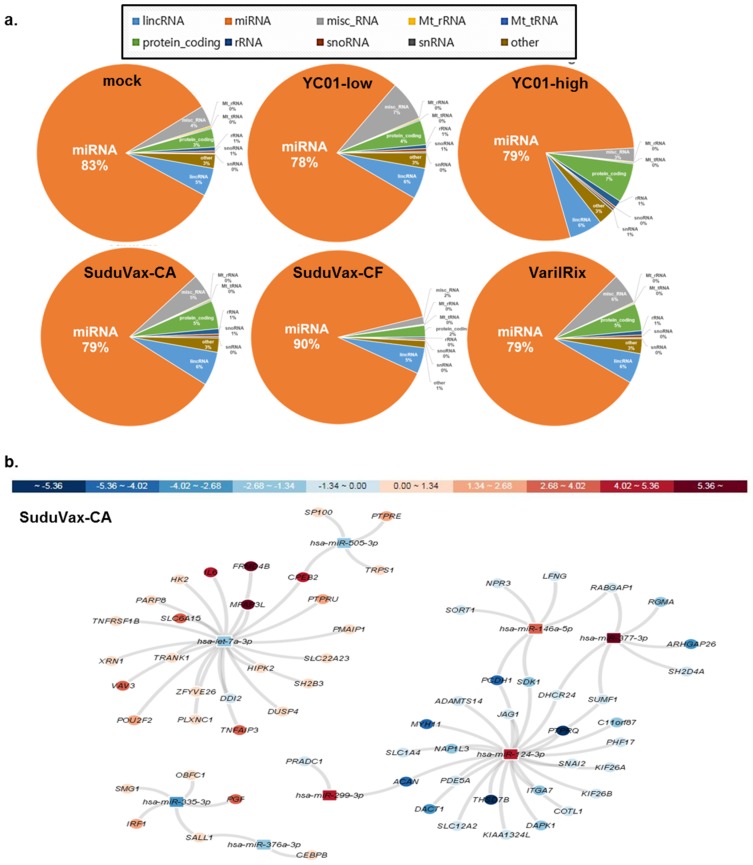
Cellular small RNA signatures in response to VZV infection in HDFs. (**a**) Distribution of mappable small RNAs by small RNA next-generation sequencing in VZV-infected HDFs. The pie chart indicates the distribution of small RNA-seq reads across the categories of annotated small RNAs (linc RNAs, miRNA, snoRNAs, tRNAs, rRNAs) in mock-treated or VZV-infected cells at 48 hpi. (**b**) miRNA–mRNA-associated network of SuduVax CA-infected HDFs. The network was resolved using a Bayesian network learning strategy and visualized using Cytoscape software. miRNA (rectangles) and their predicted target mRNAs (circles) are connected by lines. The color of each miRNA and mRNA represents their Pi score. Hubs were devised on the basis of miRNAs sharing multiple common mRNA targets.

Considering that the infection of HDFs with VZV induces a range of transcriptional changes in host mRNAs and microRNAs (miRNAs) regulating host gene expression, we used next-generation sequencing to analyze the small RNA fractions of VZV-infected cells. Distinct sets of miRNAs were found to be increased or decreased during infection. The miRNA transcriptome statistics are presented in Supplementary Table S3. In the small RNA-seq data set, several classes of small RNAs were detected including long noncoding RNAs (lncRNAs) and miRNAs (Figure 4a). A list of the miRNAs significantly dysregulated at 48 hpi was constructed using two criteria: log_2_-fold change < ±0.3 and a nominal *p*-value cut-off of 0.05 compared to mock-infected miRNA samples. The 10 most upregulated and downregulated miRNAs are presented in Supplementary Tables S4 and S5, respectively, however only a few miRNAs were highly expressed. Six miRNAs were commonly and similarly dysregulated upon VZV infection; miR-124-3p, miR-146a-5p, miR-299-3p, let-7a-3p, miR-505-3p, and miR-335-3p. Among these, miR-124-3p, miR-146a-5p, and miR-299-3p were significantly upregulated and let-7a-3p, miR-505-3p, and miR-335-3p were significantly downregulated.

**Figure 5:**
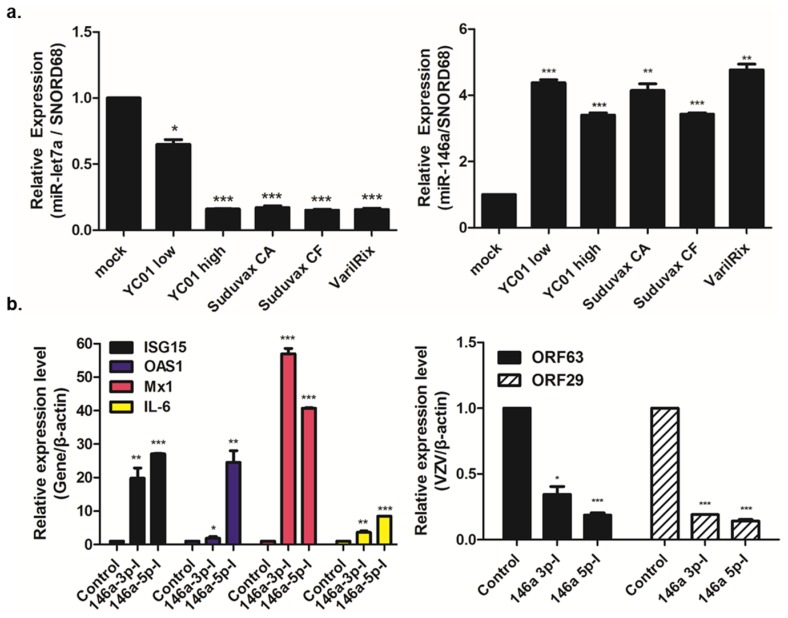
miR-146a inhibition results in a reduction of VZV gene expression. (**a**) RT-qPCR validation of miR-let-7a and miR-146a. Fold-changes are relative to mock-infected HDFs. * *p* < 0.05; ** *p* < 0.01; *** *p* < 0.001 compared with mock-infected cells. (**b**) Anti-miR-146a inhibitor (146a-3p-I or 146a-5p-I) or anti-miR negative control inhibitor (control) was transfected into HDFs. Approximately 24 h after treatment, cells were infected with SuduVax at an MOI of 0.1. RT-qPCR was performed to measure the mRNA levels of *ISG-15*, *OAS1*, *Mx1*, *IL-6*, VZV *ORF29*, and VZV *ORF63*. Target gene expression was normalized to that of β-actin mRNA and was expressed as a fold-change relative to the expression levels in control-treated cells. * *p* < 0.05; ** *p* < 0.01; *** *p* < 0.001, compared with the control-treated cells.

In order to predict the potential targets of the above miRNAs modulated by VZV, the interactive visual analysis tool miRTarVis+ was used on the miRNA–mRNA expression profile data. Figure 4b shows the image of miRNA–mRNA-associated networks between reliably differentially regulated miRNAs and host mRNA target genes. To validate the small RNA-seq results, we performed a comparative RT-qPCR analysis for two microRNAs (miR-146a-5p and let-7a-3p) using specific primers with the same experimental conditions (0.01 MOI; 48 hpi). For each miRNA, we determined the mean fold-change from three independent experiments compared with that of the mock-infected controls, and performed statistical analysis (Figure 5a). Let-7a was significantly downregulated in cells infected with attenuated VZV and wildtype YC01-low, in accordance with the results of the miRNA analysis. Meanwhile, miR-146a was consistently upregulated in both wildtype and attenuated VZV-infected cells, in accordance with the small RNA-seq data. These results support the small RNA-sequencing results and validate the cellular miRNA signature of VZV infection determined by our global approach.

To determine the role of miR-146a in the viral cycle of VZV, we used the commercially available anti-miR-146a-3p or anti-miR-146a-5p inhibitors. HDF cells were transfected with either the anti-miR-146a inhibitor (146a-3p-I or 146a-5p-I) or an anti-miR miRNA negative control inhibitor (control). The treated cells were then infected with SuduVax at an MOI of 0.1, and total RNA was isolated at 24 hpi to measure the expression levels of both VZV and host mRNAs via RT-qPCR. The inhibition of miRNA-146a increased the expression of antiviral genes, such as *ISG15, OAS1,* and *Mx1*, compared with the control-treated cells. We also confirmed that miR-146a inhibitor treatment significantly increased the expression level of *IL-6*, a known target of miR-146a, and reduced the expression levels of the VZV genes, *ORF29* and *ORF63*, compared to the control (Figure 5b).

## 4. Discussion

In this study, we conducted a comprehensive transcriptome analysis of VZV-infected cells and showed that that the most highly regulated DEGs are involved in several biological pathways related to antiviral innate immunity and inflammatory responses. Moreover, small RNA sequencing identified that several miRNAs were upregulated, including miR-146a-5p, which has been associated with other herpes virus infections, whereas let-7a-3p was downregulated in both wildtype and attenuated VZV-infected cells.

Because the immunological differences between wildtype (clinical isolates) and attenuated (vaccine) VZV strains remain unclear, we used whole transcriptome analysis to evaluate the features underlying the host immune response to VZV in a cellular model. Our data showed the distinct changes in the expression patterns of host genes in wildtype VZV-infected HDFs compared with attenuated VZV-infected HDFs. Additionally, RT-qPCR was performed to validate the RNA-seq data, which showed that attenuated VZV infection significantly upregulated the transcript levels of IFN-regulated genes such as *DDX58*, *IFIH1*, *IFN-β*, *ISG-15*, *OAS1*, and *IP-10* (Figure 3). These data support the RNA-seq data, in which significantly upregulated DEGs were mostly associated with antiviral signaling pathways, and attenuated VZV-infected cells expressed higher levels of chemokines and interferon-stimulated genes (ISGs) than wildtype VZV-infected cells. In contrast, *ORF63* transcript levels were significantly elevated in YC01-low-infected HDFs compared to attenuated VZV-infected HDFs. It remains unclear whether wildtype VZVs could replicate more efficiently and sooner post-infection, which would result in a decreased number of DEGs being involved in host antiviral innate immunity. Notably, when we analyzed the DEGs found only in the infected groups and not the mock-infected group, few interesting novel genes or genes with unknown function in response to VZV infection were found. Additionally, genes such as *DDX58*, *IFIH1*, *IFI44*, and *IFITM1* were only upregulated in the attenuated VZV-infected cells. Therefore, further studies are necessary to investigate the role of these genes in immunogenicity generation in VZV vaccines. Moreover, given that our study only included a single biological replicate of each sample for sequencing and analyzing, it remains probable that the addition of more biological replicates would increase the sensitivity towards number of DEGs identified.

We previously reported vaccine-type mutations associated with extensive in vitro serial passaging in VZV clinical strains, and there were seven vaccine-associated SNPs accumulated in highly passaged YC01 strains [12,20]. Interestingly, almost 40% of vaccine-type mutations were detected in *ORF62*, indicating that functional alterations of *ORF62* caused by these mutations may be essential for viral attenuation. Given that *ORF62* encodes for immediate early protein 62, which enables the activation of the majority of VZV promoters during lytic infection, *ORF62* mutations may regulate the expression of many VZV genes. In fact, Ko et al. previously reported that IE62 from SuduVax strains showed significantly reduced transactivation activity for the *ORF4*, *ORF28*, *ORF29*, and *ORF68* promoters compared with wildtype IE62 from wildtype VZV strains [31]. Furthermore, IE62 suppresses innate immunity by inhibiting IRF expression as well as blocking the phosphorylation of IRF3 and inhibiting IFN production [32]. Therefore, it is highly possible that the functional loss of virulence factors such as *ORF62* in attenuated VZV may contribute to the virus not being able to nullify the interferon response.

VZV infection is known to alter miRNA expression in the serum of herpes zoster patients [33,34]. We used small RNA-sequencing to detect miRNAs without the need for annotation and extended quantification over a wider dynamic range. Here, we focused on a few miRNAs commonly dysregulated by VZV infection. Among these, miRNA-146a was found to be upregulated in both wildtype and attenuated VZV-infected cells. miRNA-146a is known to be involved in the negative regulation of immune responses via the inhibition of *TRAF6* and *IRAK1*, which mediate toll-like receptor (TLR) signaling and lead to NF-κB activation [35]. Several groups have investigated the role of miR-146a in viral infection, however the antiviral role of miR-146a was shown to differ depending on the virus. miR-146a had antiviral properties against influenza infection [36], whilst it led to the regulation of *ISG* expression (including *ISG16*, *ISG56*, *OAS1*, and *Mx1),* enhanced viral replication in human cytomegalovirus-infected cells [37], and promoted the viral replication of Hendra virus and hepatitis C virus (HCV) [38,39]. The upregulation of miR-146a-5p enabled HCV-infected cells to promote liver inflammation and disease progression, and to escape from immune surveillance mechanisms, thus facilitating infection. Similarly, our results indicate that blocking miR-146a reduces VZV gene expression in vitro and suggest that this miRNA may have a positive role in VZV replication. Considering the accumulating evidence that miR-146a may be a crucial modulator of viral pathogenesis, further studies are necessary to understand the molecular mechanisms through which miR-146a mediates cellular responses to VZV.

To date, limited data has been published regarding the Korean vaccine strain SuduVax [10,11,31]. The comparative genome sequencing of clinical and vaccine strains of VZV revealed 24 vaccine-specific SNP sites that we previously detected in high passage clinical strains, such as Ellen, 32p72, YC01, and YC02 [20]. In particular, YC01-high (passage number 110) had seven SNPs, with five SNPs in *ORF62*. Given that 9 of 24 vaccine-type mutations found in multiple VZV vaccine strains were due to *ORF62*, these findings suggest that the YC01-high strain is genetically similar to the other live attenuated vaccines. This study also indicates that the host mRNA and miRNA expression signatures of YC01-high-infected cells are similar to those of the attenuated VZV strains such as SuduVax and VarilRix.

It is also worth pointing out that we included both cell-associated and cell-free forms of SuduVax in attenuated VZV groups. Although VZV is a highly cell-associated virus infecting cultured cells such as fibroblasts, it has been shown that cell-free VZVs are released from the infected dorsal root ganglia (DRG) [40]. Due to the technical challenges in obtaining sufficient yields of infectious cell-free VZV, it was difficult to compare the cellular responses of cell-free and cell-associated VZV. Here, we reported that the host transcriptome signatures caused by cell-free SuduVax and cell-associated SuduVax were quite similar. It should be pointed out that that initial infection kinetics may differ for cell-free vs. cell-associated virus infection and in particular, cell-free virus infections are established as considerably delayed compared to cell-associated virus infections. Therefore, considering the importance of the DRG in VZV latency and reactivation, further analysis of the transcriptomic differences between cell-associated and cell-free VZV infections in a cultured human DRG model would be beneficial.

Collectively, our results have generated a list of differentially expressed miRNAs and miRNAs that includes important regulators of VZV-host interactions. Among these, miRNA-146a was upregulated during VZV infection like in other herpesvirus infections, and it also affected viral gene expression. Thus, targeting miR-146a may represent a potential therapeutic tool for VZV infection. Furthermore, global transcriptomic analysis studies on VZV-infected and VZV-vaccinated humans will provide valuable insights into the VZV vaccine-induced protective immune responses.

## Figures and Tables

**Table 1:** Virus-specific DEGs including those upregulated only in wildtype and attenuated strains.

Rank	DEGs upregulated only in wildtype VZV	DEGs upregulated only in attenuated VZV
1	*AKNAD1*	*BST2*
2	*ANKRD26P1*	*CD74*
3	*DNAH12*	*CFB*
4	*FAM71F1*	*COX6B2*
5	*GAL3ST1*	*CTSS*
6	*GALNT3*	*DDX58*
7	*GRIP2*	*DLL4*
8	*IFIT2*	*ICAM1*
9	*INPP5J*	*IFI44*
10	*KBTBD8*	*IFIH1*
11	*LAT2*	*IFITM1*
12	*MYO5C*	*IL8*
13	*NEB*	*ISG20*
14	*PLEKHA6*	*LINC00475*
15	*SEMA4D*	*LYPD3*
16	*SLC6A13*	*ODF3B*
17	*ZBTB20*	*PLSCR1*
18		*RTP4*
19		*TNFAIP6*
20		*TYMP*

## Supplementary Materials

The following are available online at https://www.mdpi.com/2076-0817/8/4/183/s1, Figure S1: Biological gene ontology analysis of the gene expression profiles of VZV-infected cells, Table S1: Top 20 upregulated differentially expressed genes (DEGs) in VZV-infected cells, Table S2: Top 10 KEGG pathways enriched in upregulated differentially expressed genes (DEGs) in VZV-infected cells, Table S3: Sequencing statistics of the miRNA transcriptome, Table S4: Top 20 upregulated miRNAs in VZV-infected cells, Table S5: Top 20 downregulated miRNAs in VZV-infected cells.
