# Supplementary material for: Whole Transcriptome Analyses Reveal Differential mRNA and microRNA Expression Profiles in Primary Human Dermal Fibroblasts Infected with Clinical or Vaccine Strains of Varicella Zoster Virus
Source: Pathogens. 2019 Oct 10;8(4):183. doi: 10.3390/pathogens8040183 (PMC6963325; doi:10.3390/pathogens8040183)
Supplement: Supplementary file 1 [file pathogens-08-00183-s001.pdf]

Supplementary Figure 1. Biological gene ontology analysis of gene expression profiles of VZV-infected cells.

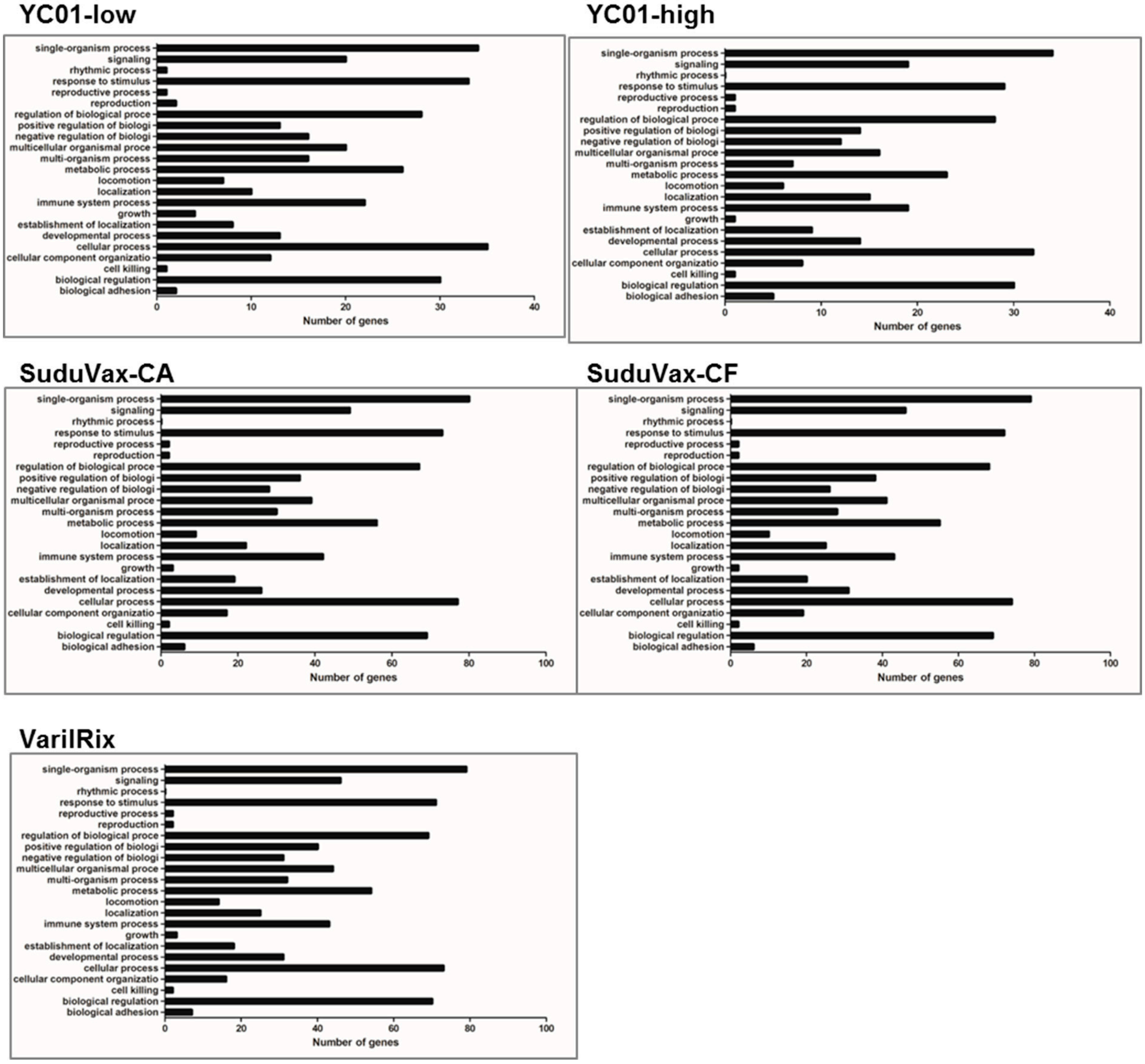

Supplementary Figure 2. Viral gene expression profiles of VZV-infected cells are shown.

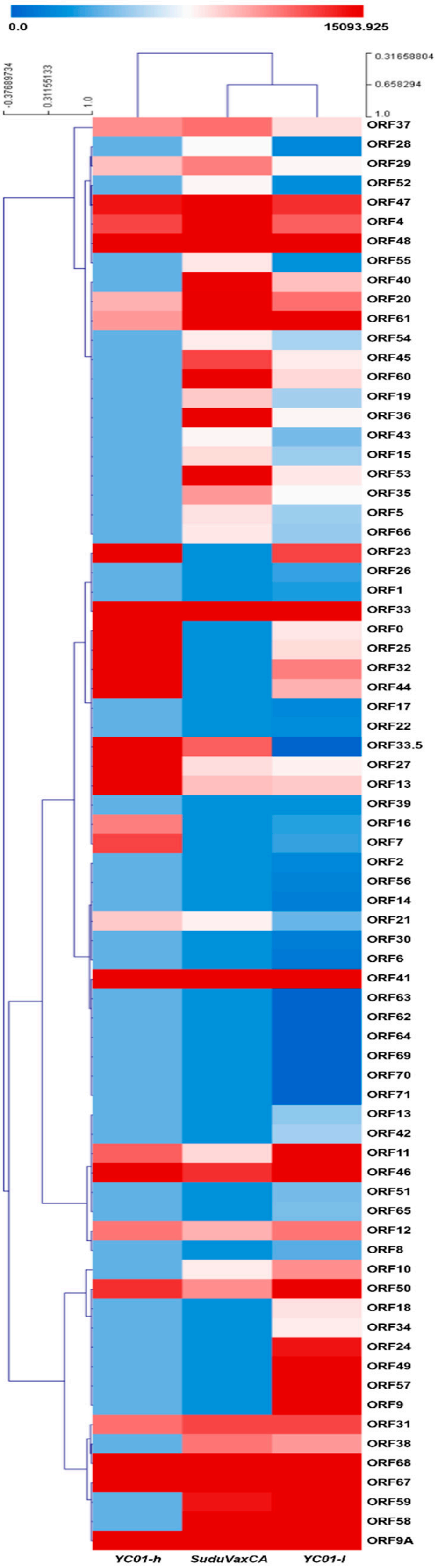

Supplementary Table 1. Top 20 up-regulated differentially expressed genes (DEGs) in VZV-infected cells.

Rank	YC01 low (DEG1)		YC01 high (DEG2)		Suduvax CA (DEG3)		Suduvax CF (DEG4)		VarilRix (DEG5)	
	Gene Name	FC	Gene Name	FC	Gene Name	FC	Gene Name	FC	Gene Name	FC
1	ANKRD26P1	188.7065	ISG15	685.0189	RSAD2	903.8879	RSAD2	916.5057	RSAD2	652.5750
2	OAS1	130.6896	IFI6	484.3815	OAS1	508.4634	OASL	347.2907	IL8	333.1435
3	CMPK2	71.0125	RSAD2	377.4129	COX6B2	487.7507	OAS1	340.1436	OAS1	282.0877
4	RSAD2	65.3448	IFI30	377.4129	OASL	349.7063	CMPK2	240.5178	OASL	274.3740
5	OASL	59.3016	C3	229.1264	CMPK2	240.5178	IL8	203.6573	CMPK2	238.8564
6	IFI44L	54.9482	BST2	191.3407	IL8	209.3829	IFI44L	140.0696	IL24	157.5865
7	GRIP2	46.5271	IFI44L	155.4169	IFI44L	149.0859	TBXAS1	108.3834	IFI44L	134.3637
8	CXCL1	44.6318	ISG20	137.1870	MX1	106.8913	MX1	106.1529	MX1	95.6704
9	MX1	44.0173	LINC00475	112.9860	IFI6	84.4485	IFI6	90.5097	IFI6	86.2229
10	ZC3H12D	34.5353	MX1	110.6608	HERC5	78.7932	HERC5	81.5719	CXCL1	81.0084
11	GAL3ST1	34.5353	TNFSF13B	101.1253	TNFSF10	78.2490	TNFSF10	73.5167	HERC5	77.7085
12	AKNAD1	29.4460	IFI27	96.3358	CXCL1	61.8199	CXCL1	58.0812	TNFSF10	64.4452
13	ZBTB20	27.8576	MEGF11	81.5719	C3	60.9688	IL24	54.5686	ROBO4	51.9842
14	TNFSF10	23.2636	MTCP1	77.7085	IL24	60.5477	IFIT1	54.1917	IFIT1	47.1766
15	KBTBD8	23.1029	OAS1	75.5835	TBXAS1	57.2816	GBP5	49.8665	LYPD3	46.5271
16	IFI6	22.0087	IFITM1	75.0614	GBP5	54.9482	C3	45.2548	GBP5	45.5696
17	ISG15	21.4068	HERC5	72.0037	IFIT1	53.0765	MX2	43.7133	C3	41.9326
18	C3	21.2590	ETV7	71.0124	IL4I1	46.2057	IFIT3	43.7133	LINC00475	39.3966
19	GALNT3	20.6777	CCDC88B	66.7178	MX2	43.7133	ETV7	39.9466	MX2	39.1245
20	DNAH12	19.9733	RTP4	64.4452	IFIT3	41.6429	ISG15	38.5859	IFIT3	37.5307

Supplementary Table 2. Top 10 KEGG pathways enriched in up-regulated differentially expressed genes(DEGs) in VZV-infected cells.

**Rank**	**YC01 low (DEG1)**		**YC01 high (DEG2)**		**Suduvax CA (DEG3)**		**Suduvax CF (DEG4)**		**VarilRix (DEG5)**	
	**Name**	**# of overlapping genes**	**Name**	**# of overlapping genes**	**Name**	**# of overlapping genes**	**Name**	**# of overlapping genes**	**Name**	**# of overlapping genes**
1	RIG-I Like Receptor signaling pathway	4/71	RIG-I Like Receptor signaling pathway	12/71	RIG-I Like Receptor signaling pathway	6/71	RIG-I Like Receptor signaling pathway	6/71	RIG-I Like Receptor signaling pathway	7/71
2			Cytosolic DNA sensing pathway	8/56	Cytosolic DNA sensing pathway	5/56	Cytosolic DNA sensing pathway	5/56	Cytosolic DNA sensing pathway	5/56
3			Phagosome	22/153	Hepatitis C	4/1314	Phagosome	5/153	Hepatitis C	5/134
4			Hepatitis C	11/134	Phagosome	4/153	Hepatitis C	5/134	Toll-Like Receptor signaling pathway	4/102
5					Antigen processing and presentation	3/76	Antigen processing and presentation	4/76	Small cell lung cancer	3/85
6					Coagulation and complement cascades	3/69	Coagulation and complement cascades	4/69	Coagulation and complement cascades	3/69
7					Toll-Like Receptor signaling pathway	3/102	Toll-Like Receptor signaling pathway	3/102		
8					Pyrimidine metabolism	3/99	Pyrimidine metabolism	3/99		
9							*Staphylococcus aureus* infection	3/55		

Supplementary Table 3. Sequencing statistics of miRNA transcriptome.

Sample	Total reads	Passing Filters (%)	Aligned Reads (%)	Precursor miRNA reads	Mature miRNA reads	Known precursor with >= 5x coverage	No. known miRNA	No. novel miRNA
**mock**	14316688	98.50	92.75	14822	10184890	869	648	233
**Yc01 low**	18805363	99.12	83.39	33325	11717928	877	644	244
**Yc01 high**	15040913	98.62	74.04	23950	16395873	934	523	211
**Suduvax CA**	17369308	98.81	72.13	36553	16791208	897	528	189
**Suduvax CF**	14129926	95.18	76.91	26210	8848023	804	495	170
**VarilRix**	13942463	99.02	73.39	22035	7942560	836	497	201

Supplementary Table 4. Top 10 up-regulated miRNAs in VZV-infected cells.

Rank	YC01 low (DEG1)		YC01 high (DEG2)		Suduvax CA (DEG3)		Suduvax CF (DEG4)		VarilRix (DEG5)	
	**miRNA**	**FC**	**miRNA**	**FC**	**miRNA**	**FC**	**miRNA**	**FC**	**miRNA**	**FC**
1	hsa-miR-6087	6.0901	hsa-miR-4697-3p	6.2169	hsa-miR-377-3p	6.0686	hsa-miR-4792	6.6715	hsa-miR-3614-3p	6.3591
2	hsa-miR-1302	6.0661	hsa-miR-4792	5.9274	hsa-miR-4697-3p	5.8510	hsa-miR-4697-3p	6.5390	hsa-miR-3614-5p	4.7556
3	hsa-miR-6724-5p	5.7279	hsa-miR-4658	5.8950	hsa-miR-3614-3p	5.5946	hsa-miR-6087	5.5251	hsa-miR-6087	4.4906
4	hsa-miR-215-5p	3.4594	hsa-miR-6087	4.9473	hsa-miR-486-3p	5.1617	hsa-miR-378d	4.7614	hsa-miR-378c	2.6749
5	hsa-miR-3180	2.3330	hsa-miR-30b-3p	4.7575	hsa-miR-6087	5.1321	hsa-miR-3614-5p	4.6765	hsa-miR-146a-5p	2.5723
6	hsa-miR-3180-3p	2.3330	hsa-miR-3614-5p	4.6835	hsa-miR-3614-5p	4.5946	hsa-miR-7641	3.7155	hsa-miR-299-3p	2.5037
7	hsa-miR-7641	2.3002	hsa-miR-124-3p	3.0205	hsa-miR-4448	3.2992	hsa-miR-1290	3.4063	hsa-miR-7641	2.3120
8	hsa-miR-1275	2.1325	hsa-miR-299-3p	2.9670	hsa-miR-7641	3.2397	hsa-miR-124-3p	3.3081	hsa-miR-361-3p	1.7465
9	hsa-miR-146a-5p	1.7578	hsa-miR-3960	2.4231	hsa-miR-124-3p	2.9531	hsa-miR-299-3p	2.9822		
10	hsa-miR-200c-3p	1.4396	hsa-miR-146a-5p	2.3629	hsa-miR-299-3p	2.7484	hsa-miR-3960	2.8633		
11	hsa-miR-182-5p	1.3421	hsa-miR-500b-5p	2.1259	hsa-miR-625-5p	2.6829	hsa-miR-1246	2.7787		
12	hsa-miR-7-5p	1.3077	hsa-miR-7641	2.1162	hsa-miR-146a-5p	2.5986	hsa-miR-146a-5p	2.2073		
13	hsa-miR-4521	1.1825	hsa-miR-361-3p	2.0419	hsa-miR-500b-5p	2.0842				
14	hsa-miR-192-5p	1.0042			hsa-miR-1246	1.8698				
15	hsa-miR-194-5p	1.0025			hsa-miR-361-3p	1.8227				
16	hsa-miR-218-5p	0.6042								
17	hsa-miR-155-5p	0.4438								

Supplementary Table 5. Top 10 down-regulated miRNAs in VZV-infected cells.

Rank	YC01 low (DEG1)		YC01 high (DEG2)		Suduvax CA (DEG3)		Suduvax CF (DEG4)		VarilRix (DEG5)	
	**miRNA**	**FC**	**miRNA**	**FC**	**miRNA**	**FC**	**miRNA**	**FC**	**miRNA**	**FC**
1	hsa-miR-6511a-5p	-7.0444	hsa-miR-335-3p	-2.9126	hsa-miR-548ap-3p	-5.6838	hsa-miR-335-3p	-2.5491	hsa-miR-548ap-3p	-5.5308
2	hsa-miR-6511b-5p	-1.6724	hsa-miR-337-3p	-2.4508	hsa-miR-548aa	-4.0127	hsa-let-7a-3p	-2.5441	hsa-miR-98-3p	-4.1923
3	hsa-miR-204-5p	-1.0769	hsa-let-7a-3p	-2.3983	hsa-miR-548t-3p	-4.0127	hsa-miR-1	-2.3128	hsa-miR-335-3p	-3.0351
4	hsa-miR-4286	-0.9624	hsa-miR-654-3p	-2.1333	hsa-miR-1296-5p	-3.1603	hsa-miR-654-3p	-2.3021	hsa-miR-1296-5p	-2.5527
5	hsa-miR-27a-5p	-0.9313	hsa-miR-450b-5p	-1.7374	hsa-miR-335-3p	-2.3204	hsa-miR-337-3p	-2.0396	hsa-miR-654-3p	-2.2961
6	hsa-miR-424-3p	-0.7373	hsa-miR-432-5p	-1.7186	hsa-miR-654-3p	-2.2503			hsa-let-7a-3p	-2.1960
7	hsa-let-7b-3p	-0.6344	hsa-miR-505-3p	-1.6505	hsa-miR-337-3p	-2.0800			hsa-miR-1304-3p	-2.1147
8	hsa-miR-1260b	-0.5961	hsa-miR-145-3p	-1.5598	hsa-let-7a-3p	-2.0773			hsa-miR-337-3p	-2.0525
9	hsa-miR-1260a	-0.5950			hsa-miR-505-3p	-1.7966			hsa-miR-1	-2.0501
10	hsa-miR-23b-3p	-0.4844			hsa-miR-744-5p	-1.7496			hsa-miR-505-3p	-1.8437
11	hsa-miR-145-3p	-0.4757			hsa-miR-376a-3p	-1.6614			hsa-miR-145-3p	-1.8157
12	hsa-miR-574-3p	-0.4489								
